# Vector Potential Index: Bridging Competence and Contribution as an Integrative Measure of Relative Transmission Capability

**DOI:** 10.1002/ece3.72705

**Published:** 2026-01-04

**Authors:** Amely M. Bauer, Nathan D. Burkett‐Cadena, Lawrence E. Reeves, Barry W. Alto, Lindsay P. Campbell

**Affiliations:** ^1^ Florida Medical Entomology Laboratory, Department of Entomology and Nematology, IFAS University of Florida Vero Beach Florida USA; ^2^ CASUS ‐ Center for Advanced Systems Understanding Helmholtz‐Zentrum Dresden‐Rossendorf e.V. (HZDR) Görlitz Germany; ^3^ Department of Community Ecology Helmholtz Centre for Environmental Research – UFZ Halle (Saale) Germany

**Keywords:** arthropod vector, host use, mosquito, surveillance, vector competence, zoonotic vector‐borne disease

## Abstract

Vector‐borne diseases (VBD) pose a major concern for public health worldwide. Identifying putative vectors and their potential contribution to transmission is a crucial step in understanding vector‐borne disease hazard. However, existing metrics are limited in their utility to inform transmission hazard in zoonotic multi‐vector, multi‐host VBD systems. We present the Vector Potential Index (VPI), a novel metric for evaluating and comparing the potential of blood‐feeding arthropod vectors to contribute to zoonotic VBD transmission. Taking a meta‐analysis approach, the VPI combines vector competence and host use data obtained from scientific literature to assign relative and absolute VPI ranks across species and transmission cycles. Using West Nile virus (WNV) in the eastern United States as a model system, our results demonstrate the ability of VPI to provide a representative assessment of vector species' potential contribution to transmission hazard in the epizootic and enzootic transmission cycles. Most species had low vector potential, and although *Aedes* species were the most competent WNV vectors in the laboratory, only *Culex* species were assigned higher VPI ranks. Additionally, the VPI suggests that the contribution of *Culex salinarius* to WNV transmission in the U.S. may be greater than previously assumed based on assessments of individual parameters. Relative and absolute VPI ranks assigned to species aligned with recent work reviewing their role as vectors in the transmission cycles, indicating that by jointly considering vector competence and host use, the VPI effectively quantifies the species‐specific potential to contribute to WNV transmission hazard in the natural environment, using existing data. We propose the objective and reproducible VPI as a powerful yet simple tool for scientists and public health practitioners, where this trait‐based approach has considerable potential to provide new insights into disease systems and enhance VBD surveillance and intervention strategies.

## Graphical abstract

The Vector Potential Index (VPI) is a novel metric for evaluating and comparing the potential of blood‐feeding arthropod vectors to contribute to zoonotic vector‐borne disease transmission. Taking a meta‐analysis approach, the VPI combines vector competence and host use data obtained from scientific literature to assign relative and absolute VPI ranks across species and transmission cycles. We propose the objective and reproducible VPI as a powerful yet simple tool for scientists and public health practitioners, where it has considerable potential to provide new insights into disease systems and enhance vector‐borne disease surveillance and intervention strategies.

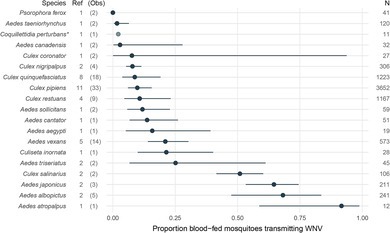

## 1 Introduction

Vector‐borne diseases are (re‐)emerging at an increasing rate, making them a major concern for public health worldwide (Dahmana and Mediannikov 2020; Kilpatrick and Randolph 2012). These diseases are caused by pathogens such as viruses and parasitic organisms, including *Plasmodium* species and filarial worms, which are transmitted by blood‐feeding arthropod vectors (Chala and Hamde 2021). Transmission of VBDs requires competent vectors, susceptible hosts, and the pathogen to overlap in space and time in a permissive environment in order for the pathogen to be maintained and amplified in the natural environment (Pavlovsky 1966). These requirements mean that it is possible to leverage biological traits, specifically vector competence and host use, to identify putative vectors and assess their potential contribution to VBD transmission cycles (Barnett 1960). Trait‐based approaches have been shown to provide a powerful framework for advancing ecological understanding of complex ecosystem functioning and have become increasingly important in community ecology. However, in the field of vector‐borne disease ecology, traits, and vector host use in particular are largely underused in quantitative frameworks to inform transmission hazard (Chandrasegaran et al. 2020; Johnson et al. 2015). Assessing traits is particularly important in zoonotic VBD systems, which often involve multiple hosts and vector species (Ferraguti 2024), with natural variation in vector traits impacting pathogen circulation and transmission outcomes (Cator et al. 2020; Kilpatrick et al. 2006; McMillan et al. 2020; Muñoz et al. 2012). However, despite a large body of work assessing host use across blood‐feeding arthropod species, a simple and generalizable metric that jointly considers vector competence and host use traits to quantify a vector species' potential contribution to transmission does not currently exist. The result is a gap in the ability to quantitatively assess the potential roles of vector species across arthropod communities and disease systems. Developing such a metric provides a data‐driven tool that may offer new insights needed to understand and predict enzootic and epizootic transmission hazard measurements in multi‐vector, multi‐host systems.

Vector competence describes a species' ability to become infected and transmit a pathogen, which is typically acquired following ingestion of an infectious blood meal (Hardy et al. 1983). Although both biotic (Lewis et al. 2023) and abiotic (Alto et al. 2014; Lambrechts et al. 2011) factors influence vector competence, it is a function of the pathogen's ability to overcome midgut and salivary gland barriers (physiological and physical) to infection within a vector, enabling subsequent transmission (Franz et al. 2015; Kramer et al. 1981). While vector competence is known to vary across species, populations, individuals, and across vector‐borne disease systems (Ferraguti 2024; Kain et al. 2022), it is key for determining the potential epidemiological importance of a vector species (Barnett 1960). As a consequence, controlled laboratory studies commonly assess this trait of putative and confirmed vector species (Alto et al. 2014; Goddard et al. 2002; Kain et al. 2022; Ruybal et al. 2016). Results from these studies are frequently used to inform vector surveillance decisions and control efforts.

Like vector competence, host competence is the ability of a host to transmit new infections to vectors (Komar et al. 2003) and can vary across species and disease systems (Kilpatrick et al. 2006; Reisen et al. 2005), which highlights host use of a vector species as crucial for pathogen acquisition and transmission of zoonotic VBD systems in the natural environment (Brackney et al. 2021; Kilpatrick et al. 2006; Muñoz et al. 2012). Moreover, vector‐host associations can be a more important driver than vector competence in determining the potential role of vector species in the transmission of pathogens in multi‐vector, multi‐host disease systems (Simpson et al. 2012; Kain et al. 2021). Although the importance of accounting for host association patterns of vectors to understanding VBD systems is well recognized (Cator et al. 2020; Garrett‐Jones 1964; Kilpatrick et al. 2005; Turell et al. 2001), previous work considering this trait when evaluating the potential contribution of vector species to transmission has been primarily qualitative (Rochlin et al. 2019; Turell et al. 2002; Turell et al. 2005). Current quantitative approaches that do incorporate host association, such as the Risk index (Kilpatrick et al. 2005), vectorial capacity (Garrett‐Jones 1964), or mechanistic models (Kain et al. 2022; Thongsripong et al. 2021), often assess transmission risk in the context of single‐vector, single‐host transmission dynamics (Garrett‐Jones 1964; Kilpatrick et al. 2005; Thongsripong et al. 2021). While useful, these metrics rely on parameters derived from fine‐scale population‐specific data, such as vector abundance and infection prevalence, that are typically obtained through targeted vector surveillance efforts (CDC 2024a; Gu et al. 2008). As a result, they are particularly valuable for informing short‐term public health responses, such as control activities of target species, or the incrimination of suspected vector species (Garrett‐Jones 1964; Kilpatrick et al. 2005). However, the use of such parameters and a focus on single hosts limits the ability of these metrics to provide insights across species, transmission cycles, and disease systems that can involve multiple hosts.

Further, as research continues to add knowledge of host use and vector competence across disease systems and species, data collected using a greater variety of methods and experimental protocols become available, which can complicate synthesis across studies (Azar and Weaver 2019; Souza‐Neto et al. 2019), requiring new methods to integrate and leverage this growing body of information. Recent advances in statistical methods developed for meta‐analyses facilitate synthesis analyses of information collected using varied approaches (Sera et al. 2019). These methods, combined with continued laboratory experiments of vector competence and field studies of host use provide new opportunities to inform metrics that can generalize vector potential across species, regions, and VBD systems.

Here, we introduce the Vector Potential Index (VPI), a new metric designed to quantify a vector species' potential to contribute to enzootic and epizootic VBD transmission hazard using two key variables: (1) vector competence, measured by the proportion of blood fed vectors transmitting a pathogen, and (2) host use, measured by the proportion of identified blood meals taken from a host. This metric does not quantify actual transmission risk to humans or other vertebrates but provides a generalizable framework to incorporate both host use and vector competence traits when assessing potential importance in transmission cycles. As such, VPI complements existing indices by providing a data‐driven tool with insights that can improve general understanding of complex VBD systems and support mid‐ to long‐term surveillance and management decisions. For example, VPI may aid public health preparedness by objectively identifying potential arthropod vectors of pathogens that pose emerging threats in a region, or identify generalizable patterns that can improve predictions of transmission hazard across environments in broadly distributed, complex multi‐host, multi‐vector VBD systems. Moreover, VPI is useful for identifying quantitative changes in a vector species' potential to contribute to enzootic and epizootic transmission within geographic regions experiencing the introduction or establishment of new potential vectors or hosts (e.g., invasive species). We demonstrate the utility of the metric using WNV as a model system. West Nile virus is the most widely distributed arbovirus globally and can be transmitted by a large number of mosquito vector species that vary across regions. As a case study, we focus on the eastern U.S., which supports a diverse assemblage of potential WNV vectors. Since its introduction in 1999, WNV has become the leading cause of mosquito‐borne disease in the continental U.S. (CDC 2022c; Ronca et al. 2021). Despite significant advancements in understanding the virus ecology and transmission dynamics, and while advances in forecasting and prediction have been made, WNV remains challenging to predict (Holcomb et al. 2023; Moore et al. 2025). Taking a meta‐analysis approach, the VPI integrates vector competence and host use traits obtained from the scientific literature to rank enzootic and epizootic vector potential across a suite of species.

We expect the inclusion of host use to provide a more holistic assessment of species' actual contribution to enzootic and epizootic transmission in the natural environment. Specifically, we expect that VPI will show some mosquito species that are highly competent WNV vectors in laboratory studies are unlikely to play an important role in the enzootic and epizootic transmission cycles, while other species that are often overlooked may be ranked higher because of their host use traits. This framework addresses the limitations of current metrics by providing a hazard‐based measure that allows assessment and comparison of the potential for a vector species to contribute to a given transmission cycle, while limiting the need for population or species‐specific data to two variables. Moreover, VPI can be adapted across different indirectly transmitted disease systems, spatial scales, and temporal contexts. Consequently, the approach offers a practical tool for investigations at both species and community levels and has considerable potential for versatility to enhance surveillance and intervention strategies and in vector‐borne disease research.

## 2 Materials and Methods

### 2.1 WNV as a Study System

West Nile virus is a globally distributed mosquito‐borne *Flavivirus* (*Flaviviridae*) that circulates in a multi‐host, multi‐vector disease system transmitted through the bite of infectious female mosquitoes (CDC 2022a). The virus is naturally maintained in an enzootic cycle involving avian amplification hosts and ornithophilic mosquito vectors, primarily in the genus *Culex*, subgenus *Culex* (Kilpatrick 2011; Petersen et al. 2013). Mosquito species that feed on both avian and mammalian hosts can act as bridge vectors that facilitate occasional spillover of WNV to humans and horses, particularly when seasonal host use shifts from birds to mammals occur (Kilpatrick et al. 2006). Although most human infections are asymptomatic, WNV is the leading cause of arboviral encephalitis worldwide, with approximately 1 in 150 infections progressing to neuroinvasive disease (CDC 2022c; Petersen et al. 2013). Variation in the composition of WNV host and vector communities at the regional scale results in heterogeneous patterns of enzootic and epizootic transmission across the U.S. (Rochlin et al. 2019). Since 1999, WNV has been detected in dead birds of over 300 species (CDC 2024b) and in field collections of ~65 mosquito species (CDC 2017), of which more than 30 species have been assessed for vector competence in laboratory studies.

### 2.2 Literature Search

We conducted a comprehensive literature search using Google Scholar and Web of Science. We used combinations of trait‐specific and general search terms to identify scientific literature on WNV vector competence and vector species host use. General keywords included “mosquito” and “Culicidae”, as well as genera and species names of the 65 mosquito species testing positive in the field. No language or document type constraints were applied to capture any accessible articles and book chapters published up to October 18, 2023. Additionally, we supplemented database searches by reviewing references cited in pertinent literature.

We first screened the titles and abstracts of identified studies to exclude references not aligned with the purpose of our search. We then reviewed the remaining articles against the following general inclusion criteria: (1) reported original research or previously unpublished data, (2) specified (minimum) sample size for each reported species, (3) tested mosquito specimens originating in North America, (4) identified mosquitoes to the species level, and (5) relevant data could be extracted from tables, text, or figures.

For studies investigating WNV vector competence, our trait‐specific search terms included “West Nile virus”, “WNV”, “vector competence”, and “competency”. We also applied additional inclusion criteria to select only studies on vector competence that (1) orally exposed female mosquitoes to WNV, (2) maintained blood‐fed specimens at a temperature of 28°C ± 3°C, (3) evaluated transmission rates ~14 days post‐exposure, and (4) used virus strains documented in the U.S.

Our search for studies on vector host use was based on the keywords “host”, “host use”, “host‐use”, “blood”, “blood meal”, “blood‐meal”, “blood feeding”, and “blood‐feeding”. Additional trait‐specific inclusion criteria required that studies (1) tested blood meals of wild‐caught female mosquitoes and (2) did not use animal or human bait in their collection protocol. In line with our objective, we further limited our search to articles that (3) reported host use of species for which we identified WNV vector competence studies and (4) sampled populations within the eastern U.S. Here, we defined the eastern U.S. as the area within the Eastern Temperate Forests terrestrial ecological region of North America (Appendix 1), a broad‐scale area of general ecological similarity based on ecosystems and environmental resources defined and described by the Commission for Environmental Cooperation (CEC 1997). However, for mosquito species where sampled populations resulted in less than five publications or 100 identified blood meals, we expanded our search to include host use data collected in other areas of the U.S.

### 2.3 Data Acquisition and Preparation

We created two datasets, one for each trait, by reviewing articles and Supporting Information from studies that met our inclusion criteria. For each study and mosquito population tested, we cataloged the scientific name to the highest reported taxonomic resolution, sample size, spatiotemporal information on mosquito sampling and experimental design, and any reported trait‐specific variables. We verified scientific names using the “taxize” (Chamberlain and Szocs 2013) package and updated species‐level names as needed to account for changes in taxonomic identity over time and facilitate species‐level meta‐analyses. Data recorded for species hybrids or grouped species were excluded from further analyses. We then used field‐collection data from the study area, available through National Ecological Observatory Network (2024), VectorBase (2022), and GBIF (https://www.gbif.org), to identify which species to include in analyses. Importantly, we chose to exclude the laboratory‐tested vectors *Aedes dorsalis* (Meigen), *Culex stigmatosoma* Dyar, *Culex tarsalis* Coquillett, *Culex thriambus* Dyar, *and Culiseta incidens* (Thomson) from this study, even though these species occur within the study area. In the eastern U.S., they are captured infrequently and in low abundances (NEON 2024; VectorBase 2022), suggesting that occurrences in our study area lie at the very eastern extent of the species' geographic range (Rhodes et al. 2023).

To assess the potential of a species to contribute to WNV transmission, vector competence was defined as the proportion of female mosquitoes capable of transmitting the virus after ingesting an infectious blood meal. Therefore, studies were excluded if they quantified vector competence using other transmission metrics (e.g., based on infection or disseminated infection in females) without providing the data necessary to calculate the proportion of blood‐fed females transmitting WNV.

We defined host use as the class‐level host associations of an individual mosquito species, measured by the proportion of identified blood meals taken from a host class. We allowed studies reporting mixed or unidentified blood meals to be included in the dataset, while studies reporting identified blood meals only for specific taxonomic groups (e.g., within a target host class or order) were excluded. The resulting dataset of mosquito host use from the published literature was augmented by additional field‐collected blood meal records (L.E. Reeves, unpublished data). For each mosquito population tested, we aggregated all identified blood meal records at the taxonomic level of host class, that is Aves, Mammalia, Amphibia, and Reptilia. We then further aggregated reptilian and amphibian blood meal counts because here we did not consider hosts in those groups as contributing to the WNV transmission system, and because early studies often reported blood meals jointly for these classes.

Next, we determined the number of events (i.e., mosquitoes transmitting WNV or feeding on a host class) reported across studies in preparation for meta‐analyses. To address heterogeneous reporting of vector competence and host use, we then aggregated results within studies that reported multiple outcomes for a given species and trait. To mitigate sampling design differences, sample sizes and event counts were summarized by study, mosquito population (local or state level), year, and, for vector competence studies, WNV titer and temperature. Each of the aggregated outcomes was considered an individual observation in our meta‐analyses. A list of all data sources used in these analyses is provided in Appendix 2.

### 2.4 Meta‐Analyses

We performed a meta‐analysis of single proportions implementing generalized linear mixed‐effects models (GLMMs) in R (R Core Team 2024) to synthesize overall proportions of vector competence, avian host use, and mammal host use. While the approach does not return individual study weights, GLMM‐based methods have been recommended for meta‐analysis of proportions, especially when studies included in the analysis report rare events or small sample sizes, or when sample sizes across studies cover a wide range (Lin and Chu 2020; Schwarzer et al. 2019). For all analyses, proportions were calculated as the number of positive events divided by the total number tested, and then logit‐transformed prior to pooling to avoid a skewed sampling distribution that can result from their restricted range (0–1).

First, we fitted meta‐analytic multilevel GLMMs for each trait to investigate the distribution of variance across study outcomes using the “metafor” package (Viechtbauer 2010). GLMMs of logit‐transformed proportions included random effects for mosquito species and observation nested within study, which accounted for non‐independence of observations in studies that reported multiple results. Models were then used to evaluate whether the reported results were consistent within and across studies and species, as quantified by *I* ^2^. The *I* ^2^ statistic quantifies the amount of variance across effect sizes that is not attributed to sampling error (Higgins and Thompson 2002) and has been recommended to assess consistency in data used in proportional meta‐analyses (Barker et al. 2021). For each model, we computed overall *I* ^2^ as well as *I* ^2^ partitioned between random effects.

Then, we applied default settings in the “meta::metaprop()” function (Balduzzi et al. 2019) to fit random intercept logistic regression models and synthesize pooled proportions for each trait and species. A random effect at the observation level was included to account for variation within and between results included in the meta‐analyses. 95% confidence intervals of estimated overall proportions were calculated using the Clopper‐Pearson interval (Balduzzi et al. 2019).

### 2.5 Vector Potential Index

A two‐step approach was employed to characterize species‐level VPI for enzootic and epizootic transmission cycles. We first quantified the potential of a vector species to contribute to transmission (VP), then applied an equal interval scale to the calculated raw VP values to quantify ranked VPI. Categorizing the VP values into ranks enables the use of VPI as a descriptive tool that facilitates interpretation across multiple species and enzootic and epizootic transmission cycles. The VPI can be assigned as both a relative or an absolute index, in which ranks represent a scale ranging from “low potential” to “high potential” for species with VP > 0. Species with VP = 0 were considered to have “no potential” to contribute to transmission.

#### 2.5.1 Calculating VP

We used pooled proportions obtained from meta‐analyses of each trait to calculate VP as their joint probabilities as

$$\mathrm{VP}=p_{\text{vector}\;\text{competence}}\times p_{\text{host}\;\text{use}}$$

where p is the overall proportion of a given trait. For the enzootic maintenance cycle, a species' VP for WNV was calculated as

$${\mathrm{VP}}_{\text{enzootic}}=p_{\text{vector}\;\text{competence}}\times p_{\text{host}\;\text{use}\;:\;\text{avian}}$$

which ensures that species with high vector competence and frequent avian host use were assigned the highest scores. In contrast, species with low rates documented for either trait received the lowest scores. To estimate VP for the epizootic cycle of WNV, mammal host use was included in the formula:

$${\mathrm{VP}}_{\text{epizootic}}=p_{\text{vector}\;\text{competence}}\times p_{\text{host}\;\text{use}\;:\;\text{avian}}\times p_{\text{host}\;\text{use}\;:\;\text{mammal}}$$

Because host use proportions across all host classes sum to ~1, species with the greatest $p_{\text{host}\;\text{use}}$ contribution to epizootic VP was those that feed equally often on both avian and mammalian hosts. This property of $p_{\text{host}\;\text{use}}$ is designed to account for the fact that horizontal transmission (i.e., by bite) is the primary mode of infection for female mosquitoes (Lequime et al. 2016), requiring them to feed on both a maintenance and spillover host, that is birds and mammals, respectively, in the case of WNV.

#### 2.5.2 Assigning VPI

For the relative VPI, ranks are scaled from zero to the highest VP value calculated for any of the species included in a target area or specific study. Here, we used a five‐step equal interval scale to assign each species a relative VPI rank representing low (VPI_5_ = 1) to high (VPI_5_ = 5) potential for VP values greater than zero. Relative VPI was assigned for the enzootic and for the epizootic transmission cycles, relative to the species included in each analysis.

To assign absolute VPI, ranks are scaled to the highest possible VP value for a given transmission cycle (e.g., enzootic or epizootic transmission of WNV), which allows for broader comparisons across species and transmission cycles or as additional information becomes available. The highest possible VP represents an “ideal” vector species, where 100% of individuals are transmitting the pathogen and blood meals are taken at a host use ratio assumed to be optimal for transmission. For WNV transmission, the equal‐interval scale is applied to a range of values between zero and 1 (i.e., 100% of blood meals are taken from avian hosts) for enzootic transmission, and between zero and 0.25 (i.e., 50% of blood meals taken from mammalian and avian hosts, each) for the epizootic transmission cycle. Here, we used a ten‐step scale to assign each species an absolute VPI rank representing low (VPI_10_ = 1) to high (VPI_10_ = 10) potential for VP values greater than zero for enzootic and epizootic transmission cycles.

## 3 Results

### 3.1 WNV Vector Competence

**FIGURE 1:**
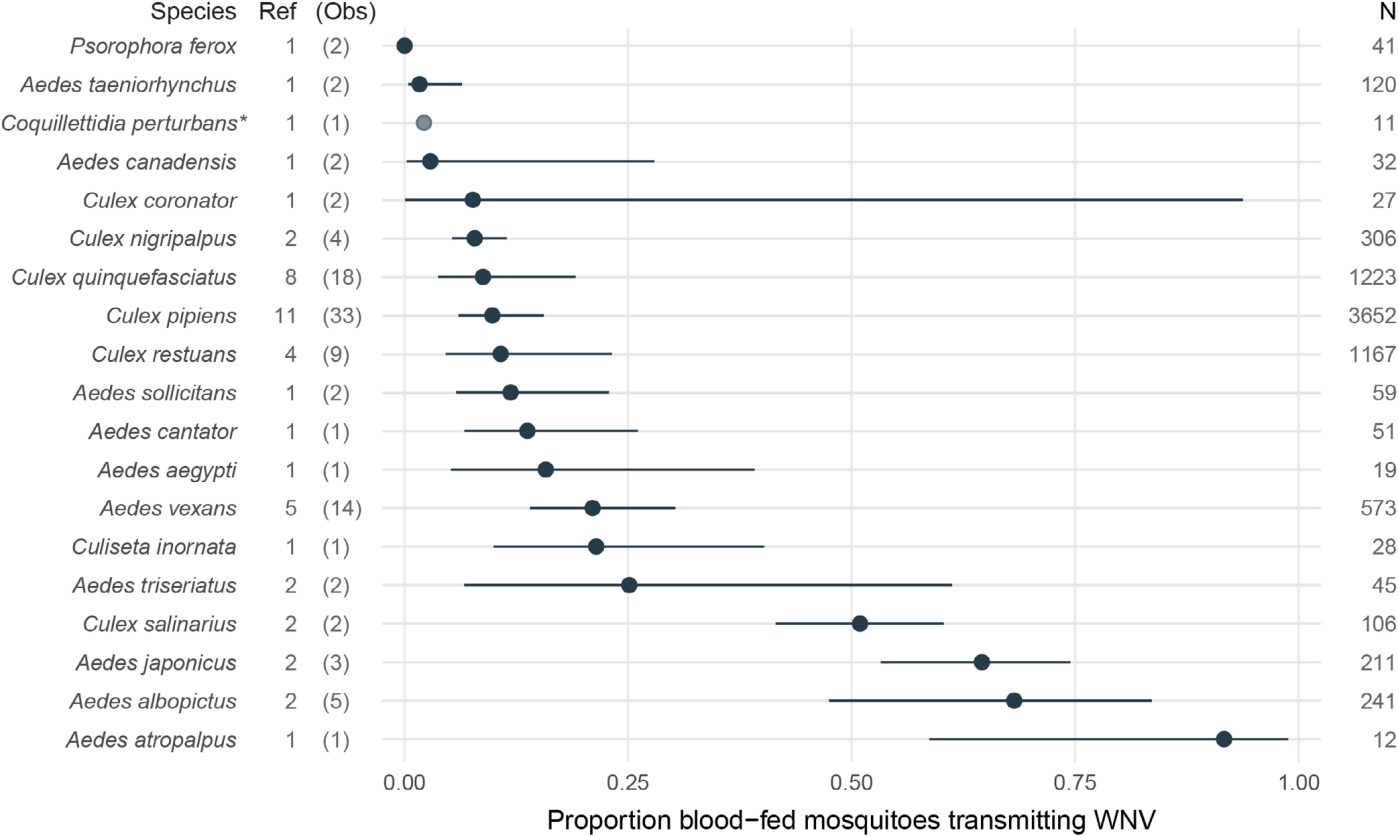
Proportional meta‐analysis of mosquito species' West Nile virus vector competence. Points represent the overall proportion of WNV transmitting female mosquitoes (arranged in ascending order) and the solid lines show the 95% confidence interval. Ref: Number of identified studies, Obs: Number of observations included in proportional meta‐analysis, N: Total number of tested specimens, *as reported in Sardelis et al. (2001).

Our literature search identified 23 published WNV vector competence studies that met our inclusion criteria and data requirements (Table A2). These articles provide data for 27 mosquito species, and we included 19 species occurring in the Eastern Temperate Forests ecoregion in the final data set. Data availability varied greatly between species. For ~80% of species, our search identified only one (*N* = 10) or two (*N* = 5) studies assessing WNV vector competence. The most studied mosquitoes were *Cx. pipiens*, *Culex quinquefasciatus* Say, *Aedes vexans* (Meigen), and *Cx. restuans*, with 11, 8, 5, and 4 publications that resulted in 33, 18, 14, and 9 observations for inclusion in meta‐analyses (Figure 1). Across species, total sample size ranged from 11 ( *Aedes atropalpus* (Coquillett)) to 3652 (*Cx. pipiens*) tested specimens.

We found that substantial variance in the vector competence data set was not attributed to chance (*I* ^2^ = 95%). Differences in tested mosquito species explained almost half of the observed variance across the data (*I* ^2^ = 45%), while between‐study and within‐study variability explained a lower amount of variance each (*I* ^2^ = 26% and *I* ^2^ = 23%, respectively). Meta‐analyses of single proportions were conducted for 18 of the 19 species included in the data set. Because the only study on *Coquillettidia perturbans* (Walker) vector competence reported a small sample size (*N* = 11) and a low estimated transmission rate (2%), we used the estimated rate reported by Sardelis et al. (2001) as a proportion of mosquitoes transmitting, rather than performing a meta‐analysis to avoid inflating vector competence values. Among the species included in meta‐analyses, the overall proportion of WNV vector competence was highest for *Ae. atropalpus* (0.92), followed by *Aedes albopictus* (Skuse) (0.68), *Aedes japonicus* (Theobald) (0.65), and *Culex salinarius* Coquillett (0.51), which was the highest ranking *Culex* mosquito (Figure 1).

### 3.2 Host Use

**FIGURE 2:**
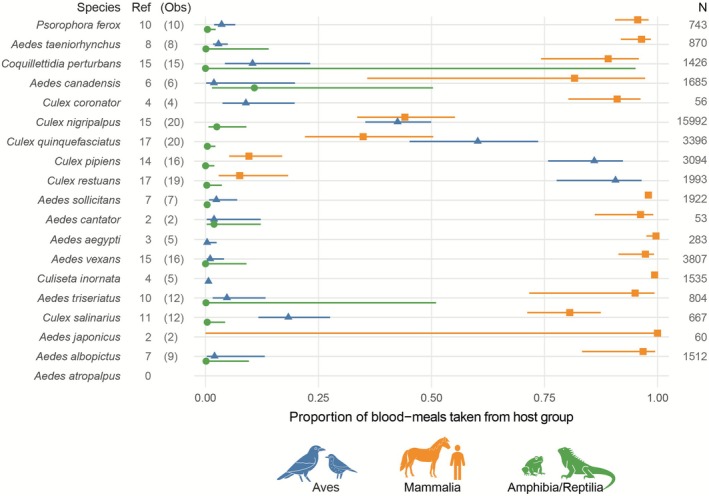
Proportional meta‐analysis of mosquito vector species host use. Species are arranged in ascending order of overall laboratory‐confirmed vector competence. Points represent the overall proportion of blood meals taken from a host group, and the solid lines show the 95% confidence interval. Ref: Number of identified studies reporting host use, Obs: Number of observations included in proportional meta‐analysis, N: Total number of tested specimens.

Data extracted from a total of 41 publications were included in the final host use data table, comprising records for 18 of the 19 species included in the WNV vector competence meta‐analyses (Table A2). Blood meal PCR results from field collections (L.E. Reeves, unpublished data) provided additional host use data for 10 species. Our search did not identify any data quantifying host use of *Ae. atropalpus*, whereas at least two records were available for all other mosquito species. Because of the low number of blood meals collected in the study area (*N* = 7), additional data collected across North America were also included for *Culiseta inornata* (Williston). Mosquito species with the greatest number of observations available for meta‐analyses were *Cx. quinquefasciatus* (*N* = 21), *Culex nigripalpus* Theobald (*N* = 20), and *Cx. restuans* (*N* = 19). The total number of blood meals from identified hosts varied between 53 ( *Aedes cantator* (Coquillett)) and 15,992 (*Cx. nigripalpus*) (Figure 2).

A substantial amount of variance in the host use data set was not attributed to chance (*I* ^2^ = 99%). For both the avian and mammalian host use subset, this variance was largely explained by differences in the mosquito species investigated (*I* ^2^ = 50% and *I* ^2^ = 53%), while between‐study and within‐study differences accounted for moderate (*I* ^2^ = 38% and *I* ^2^ = 32%) and low (*I* ^2^ = 11% and *I* ^2^ = 14%) amounts of variation, respectively. Among the included genera, *Culex* species showed the highest avian host use, particularly *Cx. restuans* (0.91) and *Cx. pipiens* (0.86). *Culex quinquefasciatus* and *Cx. nigripalpus* fed on birds and mammals almost equally, with estimated proportions of 0.61 and 0.34, and 0.43 and 0.44, respectively. Species involved in WNV vector competency studies rarely fed on reptilian or amphibian hosts, except for *Aedes canadensis* (Theobald), where ~10% of the blood meals were taken from these host classes.

### 3.3 Vector Potential Index

VPI scores revealed that 16 of the 18 mosquito species for which VP could be calculated showed at least some potential to contribute to WNV transmission, and that VPI was heterogeneous across species and WNV transmission cycles (for calculated VP values, see Appendix 3). Relative VPI was low for the majority of species (relative VPI_5_ ≤ 1), and all species with greater vector potential were members of the genus *Culex*. Species with the greatest relative potential contribution (VPI_5_ = 5) to the enzootic cycle were *Cx. pipiens* and *Cx. restuans*, as well as *Cx. salinarius*, which was also the only species with the highest possible relative epizootic vector potential (VPI_5_ = 5) (Table 1). In addition, more species were shown to have higher potential contributions (VPI_5_ > 1) in WNV enzootic cycle transmission (*N* = 5) than epizootic transmission (*N* = 2) in the study area. All included species showed a low absolute potential to contribute to WNV transmission in the enzootic and epizootic cycle (absolute VPI_10_ ≤ 1), respectively, except *Cx. salinarius*, for which absolute epizootic VPI approached a moderate vector potential (VPI_*10*_ = 4).

**TABLE 1:** Relative and absolute enzootic (enz) and epizootic (epi) WNV VPI of mosquito vector species in the Eastern Temperate Forests ecoregion of North America.

Species	Overall proportion			Relative VPI_5_		Absolute VPI_10_	
	WNV vector competence	Avian host use	Mammal host use	enz	epi	enz	epi
*Psorophora ferox*	0.000	0.036	0.956	0	0	0	0
*Aedes japonicus*	0.646	0.000	1.000	0	0	0	0
*Aedes taeniorhynchus*	0.017	0.029	0.965	1	1	1	1
*Coquillettidia perturbans*	0.022^a^	0.113	0.882	1	1	1	1
*Aedes canadensis*	0.029	0.019	0.817	1	1	1	1
*Culex coronator*	0.076	0.089	0.911	1	1	1	1
*Aedes sollicitans*	0.119	0.024	0.980	1	1	1	1
*Aedes cantator*	0.137	0.019	0.962	1	1	1	1
*Aedes aegypti*	0.158	0.004	0.997	1	1	1	1
*Aedes vexans*	0.210	0.013	0.967	1	1	1	1
*Culiseta inornata*	0.214	0.007	0.994	1	1	1	1
*Aedes triseriatus*	0.251	0.047	0.950	1	1	1	1
*Aedes albopictus*	0.682	0.020	0.968	1	1	1	1
*Culex nigripalpus*	0.078	0.425	0.441	2	1	1	1
*Culex quinquefasciatus*	0.088	0.614	0.335	3	2	1	1
*Culex pipiens*	0.098	0.860	0.096	5	1	1	1
*Culex restuans*	0.108	0.907	0.076	5	1	1	1
*Culex salinarius*	0.509	0.183	0.806	5	5	1	4
*Aedes atropalpus*	0.917						

^a^ Vector competence as reported in Sardelis et al. (2001).

## 4 Discussion

Understanding and predicting the risk of zoonotic VBD transmission is crucial for the development of effective prevention and intervention strategies. Here, we introduce the Vector Potential Index (VPI), a metric integrating vector competence and host use, two important vector traits that can change VBD dynamics by affecting transmission rates. Using WNV in the eastern U.S. as a model system, we demonstrate that VPI quantifies the species‐specific potential to contribute to transmission in the enzootic and epizootic WNV cycles, with relative and absolute VPI scores reflecting species roles reported in the literature (Rochlin et al. 2019). The VPI metric provides a powerful and flexible metric for evaluating and comparing the potential of blood‐feeding arthropod vectors to contribute to zoonotic VBD transmission.

Our results indicate that by jointly considering vector competence and host use, VPI ranks provide a realistic approximation of a mosquito species' potential to contribute to zoonotic VBD transmission in the natural environment. Specifically, we found that avian host use drove high relative enzootic VPI for both *Cx. pipiens* and *Cx. restuans*, which have been linked to intense enzootic WNV amplification in the northeastern and eastern U.S. (Kilpatrick et al. 2005; Rochlin et al. 2019). In contrast, most *Aedes* vectors in the study area obtained their blood meals from mammals. As expected, this mammalophilic host use resulted in low enzootic and epizootic VPI ranks for these vectors, despite *Aedes* species representing four of the five most competent WNV vectors in the laboratory. Overall, relative and absolute VPI scores across transmission cycles and species were consistent with previous work evaluating potential roles of individual species in the WNV disease system in North America (Molaei et al. 2006; Sardelis et al. 2001; Turell et al. 2005) and within our study area (Andreadis 2012; Rochlin et al. 2019).

However, we found that for some species, VPI ranks differed from previous assessments. In line with our expectations, VPI indicated that *Cx. salinarius* may play a more important role in WNV transmission than previously suggested. While high relative enzootic and epizootic VPI ranks due to high vector competence and more opportunistic host use are consistent with several studies that recognized *Cx. salinarius* as a locally important vector in the eastern U.S. (Godsey et al. 2012; Molaei et al. 2006), its contribution to WNV transmission is often considered limited to occasional epizootic spillover in coastal areas (Andreadis 2012; Kilpatrick et al. 2005). Interestingly, relative and absolute epizootic VPI suggest that *Cx. salinarius* has a much higher potential to contribute to WNV spillover than any other vector evaluated in this study. This result seemingly contrasts with a risk assessment by Kilpatrick et al. (2005) that suggested, due to its overall low abundance, *Cx. salinarius* may be responsible for less than 5% of human WNV infections in the northeastern U.S., while *Cx. pipiens and Cx. restuans* account for as much as 80% of spillover transmission in that area. However, it has been argued that commonly employed collection methods, such as CO_2_‐baited light traps, can significantly under‐represent *Cx. salinarius* abundances relative to observed human landing rates (Thompson 1967; Uelmen et al. 2023). In addition, morphological misidentification may further contribute to underestimated abundances and infection rates for this species (Rochlin et al. 2009; Uelmen et al. 2023). Collectively, these findings suggest that *Cx. salinarius* contribution to enzootic and epizootic transmission in the northeast may be greater than previously assumed.

A VPI ranking similar to that of *Cx. salinarius* could be expected for *Cx. tarsalis*. Although we did not consider *Cx. tarsalis* as a vector in the study area, its experimental vector competence is comparable to that of *Cx. salinarius* (Reisen et al. 2008), and around four times higher than that of the other eastern *Culex* vectors we evaluated (Appendix 4). As with *Cx. salinarius*, a high vector competence and relatively generalist host use, despite a large proportion of avian blood meals, suggest that *Cx. tarsalis* has a higher vector potential than most of the species evaluated in our study (Table A4). This assessment is supported by previous studies that implicate *Cx. tarsalis* as the primary enzootic WNV vector in the western U.S. (Turell et al. 2005), and its distribution largely correlates with the spatially aggregated pattern of WNV spillover transmission at the regional and continental scale (Dunphy et al. 2019; Sugumaran et al. 2009). Moreover, recent continental‐scale environmental niche models suggest highly suitable environments for both *Cx. salinarius* and *Cx. tarsalis* within the North American Great Plains ecoregion (Gorris et al. 2021; Rhodes et al. 2023), which is the region with the highest human WNV incidence across the U.S. (CDC 2022b; DeGroote and Sugumaran 2012; Sugumaran et al. 2009).

In contrast, we found that despite almost equal proportions of avian and mammalian host use, VPI assigned a moderate to low enzootic and epizootic vector potential to *Cx. quinquefasciatus* and *Cx. nigripalpus*. This result was somewhat unexpected, as both species have been repeatedly treated as important maintenance and spillover vectors in the southeastern U.S. (Godsey et al. 2005; Sallam et al. 2016). However, WNV transmission in the epizootic cycle in the southeast is comparatively limited (Sugumaran et al. 2009). We found that both relative and absolute VPI ranks support the hypothesis that this pattern may, in part, be explained by the relative absence of efficient epizootic vectors in this region (Gorris et al. 2023; Rochlin et al. 2019). Overall, placing VPI within the spatial context of species distributions, we find that our results are consistent with the broad‐scale patterns in WNV transmission dynamics in the eastern U.S. (Rochlin et al. 2019). In this region, community‐level relative VPI indicates a much higher potential for enzootic transmission, especially in the northern parts of the region, than for epizootic spillover. Collectively, our case study provides evidence that VPI may effectively implicate species that could be crucial to amplification and spillover, including those that may be overlooked when other metrics are applied.

One challenge in quantitatively evaluating a vector species' traits is the high degree of variability observed across studies, which can be due to trait variation in the tested population as well as differences in experimental design (Kain et al. 2022; Souza‐Neto et al. 2019). Here, we demonstrate that information synthesized across studies using meta‐analysis approaches can be used to inform vector‐borne disease ecology (Stephens et al. 2016). Moreover, we found that trait estimates obtained from proportional meta‐analysis, rather than individual study results that reflect unique population characteristics (Cator et al. 2020), are suitable for identifying generalizable patterns across putative vector species.

Our case study illustrates how the VPI can be used to evaluate and compare different mosquito species that may contribute to transmission in a multi‐vector disease system at a regional scale. The VPI is a practical and highly versatile tool that can be applied at the species, population, or community level and is useful either as a stand‐alone application or integrated with existing approaches. For example, VPI based on more restrictively selected data may provide insights at finer scales, where it could quantify seasonal and geographic variations in potential vector contributions. In disease systems in which primary amplification hosts are known, assessing VPI using host use proportions specific to these species could provide additional insights into vector potential. In addition, because of the strong spatial component in VBD transmission dynamics (Pavlovsky 1966), using VPI in conjunction with species distribution models, in particular joint species distribution models, may prove especially insightful (Bauer et al. 2024; Chandrasegaran et al. 2020). Model outcomes and prediction maps could provide data‐driven tools that help to disentangle complex disease transmission dynamics, aid in prioritizing surveillance and control efforts across landscapes, and offer new information on how VBD transmission hazards may shift as environmental conditions and vector communities change.

While the trait‐based VPI can offer valuable insights into vector contributions to transmission dynamics in VBD systems, limitations exist. Here, we assign the highest enzootic and epizootic VPI scores to species that are highly vector competent and that feed primarily on maintenance hosts or equally on maintenance and dead‐end hosts, respectively. While this resulted in low VPI ranks for the *Aedes* species included here, their potential involvement as locally important bridge vectors in periods of high enzootic transmission remains uncertain (Rochlin et al. 2019). Despite the overall very low avian host use proportions of most *Aedes* species examined here, all have tested positive for WNV in the field (CDC 2017). Work evaluating WNV host competence suggests that some mammal species may be able to develop viremia high enough to infect blood‐feeding mosquitoes (Root and Bosco‐Lauth 2019; Tiawsirisup et al. 2005; Tonry et al. 2005), and future studies could apply the VPI to investigate and compare *Aedes* species vector potential in such a mammal‐*Aedes* WNV transmission cycle. Future research could also apply the VPI to investigate and compare species' vector potential for vector‐borne pathogens with transmission cycles for which mammals are the natural hosts (such as Venezuelan equine encephalitis virus and diverse Orthobunyaviruses), other pathogens that utilize avian hosts for amplification (St. Louis encephalitis virus, and eastern equine encephalitis virus), or pathogens that utilize both birds and mammals for amplification (Japanese B encephalitis virus). The VPI model can be easily modified to accommodate different host groups.

Data availability is an important and well‐recognized constraint in trait‐based analyses (Cator et al. 2020; Kain et al. 2022), which is reflected in our study. For example, the role of *Culex coronator* Dyar and Knab, a species that has recently spread eastward into the southeastern U.S., as a vector of WNV remains currently unknown, despite repeatedly raised public health concerns (Connelly et al. 2016; Sames et al. 2021). Here, VPI indicated a low vector potential for this species. However, low VPI was driven by low vector competence, where limited data availability resulted in high uncertainty, highlighting that this result should be interpreted with caution. Moreover, species traits are known to vary within species and across environmental conditions, as well as space and time (Kilpatrick et al. 2010; Ruybal et al. 2016). Because VPI is based on estimates, the metric may quantify VBD transmission hazard less reliably for understudied species (Kain et al. 2022). In addition, our literature search did not yield information for some species, resulting in their exclusion from our analyses. Specifically, no host use data were available for *Ae. atropalpus*, the most laboratory competent WNV vector included in our study. While reported mammalophilic host use (Berry and Craig Jr. 1984) suggests that *Ae. atropalpus* has no WNV vector potential; other species implicated in WNV transmission, such as the host generalist *Culex erraticus* (Dyar & Knab) (Table A3), have not yet been tested for vector competence in laboratory experiments. Despite these limitations, insights from the associated literature search and interpreted VPI results may aid in the identification of current knowledge gaps and provide support for future data collection efforts.

Overall, our evaluation suggests that ranked VPI captured the potential of species to contribute to WNV transmission well. Currently, most trait‐based approaches remain limited to mechanistic models of single vector species (Cator et al. 2020; Ferraguti 2024). However, increasing efforts to investigate community‐level effects highlight the need for consideration of species trait variations that influence individual roles in multi‐vector transmission dynamics (Chaves et al. 2011; Cleveland et al. 2023; Johnson et al. 2012; McMillan et al. 2020; Roche et al. 2013). Here, VPI offers new opportunities for identifying generalizable patterns across vector communities and transmission cycles, even when community species membership varies across ecological conditions and scales.

## 5 Conclusion

Our VPI metric integrates differences in two vector traits that are known to influence pathogen transmission rates, providing a flexible framework for assessing and comparing the potential contributions of vectors to transmission in VBD systems. VPI has many possible applications across diverse VBDs and scales, including investigations at both species and community levels. As illustrated in our case study, the metric can focus on different vectors implicated in the transmission of a selected disease system or transmission cycle, but could also focus on individual mosquito species and their vector potential for various pathogens. We note that the VPI framework could also be easily adapted to other ecological systems, including host/parasite systems or plant/pollinator systems. Most importantly, we propose the objective and reproducible VPI as a powerful yet simple tool for scientists and public health practitioners, where this trait‐based approach has considerable potential to provide new insights into complex disease systems and enhance surveillance and intervention strategies.

## Author Contributions

**Amely M. Bauer:** conceptualization (lead), data curation (lead), formal analysis (equal), investigation (equal), methodology (lead), software (lead), validation (lead), visualization (lead), writing – original draft (lead), writing – review and editing (equal). **Nathan D. Burkett‐Cadena:** conceptualization (lead), methodology (lead), writing – original draft (supporting), writing – review and editing (equal). **Lawrence E. Reeves:** conceptualization (supporting), methodology (supporting), resources (equal), writing – original draft (supporting), writing – review and editing (equal). **Barry W. Alto:** conceptualization (supporting), methodology (supporting), writing – review and editing (equal). **Lindsay P. Campbell:** conceptualization (lead), methodology (lead), resources (equal), writing – original draft (supporting), writing – review and editing (equal).

## Conflicts of Interest

The authors declare no conflicts of interest.

## Data Availability

All data are available for download from Zenodo, https://doi.org/10.5281/zenodo.14773769. The provided files include all necessary data and scripts to replicate the presented results.

## Appendix 1

Geographic extent of the Eastern Temperate Forests terrestrial ecological region of North America. Level I ecological region shapefile available through the Commission for Environmental Cooperation's North American environmental atlas (http://www.cec.org). Background aerial map Earth Start Geographics SIO, Microsoft Corporation © 2024, available through Bing Maps and accessed through QGIS v 3.16.16.

## Appendix 2

Studies from which data were extracted for meta‐analyses. Tabular overview and list of all original data sources by vector species and trait.

**TABLE A2:** Overview of identified original data sources meeting inclusion criteria, by species and trait.

Species	Vector competence	Host use	Hose use^a^
*Aedes aegypti*	1	25–27	67
*Aedes albopictus*	1, 2	27–32	67
*Aedes atropalpus*	1		
*Aedes canadenis*	3	25, 33–37	
*Aedes cantator*	3	36, 38	
*Aedes japonicus*	1, 4	33, 36	
*Aedes sollicitans*	1	30, 33, 36, 38–41	
*Aedes taeniorhynchus*	1	33, 36, 41,^b^	
*Aedes triseriatus*	3, 5	25, 31, 33, 36, 38, 41–43,^b^	
*Aedes vexans*	1, 3, 6–8	25, 30, 31, 33, 38, 40–42, 44–48,^b^	64, 68, 69
*Coquillettidia perturbans*	9	25, 30, 33, 35–38, 40–42, 45,^b^	
*Culex coronator*	10	49, 50,^b^	
*Culex nigripalpus*	9, 11	49–55,^b^	
*Culex pipiens*	1, 6–8, 12–18	29–31, 33, 35, 37, 38, 42, 46, 47, 56–59	64, 65
*Culex quinquefasciatus*	6, 9, 14, 17–21	25–28, 40, 44, 45, 49, 50, 53–55, 59–61,^b^	68–73
*Culex restuans*	9, 15, 16, 22	25, 29–31, 33, 35, 37, 38, 42, 46, 50, 53, 56, 58, 59, 62, 63,^b^	
*Culex salinarius*	9, 23	25, 30, 33, 34, 38, 40, 42, 44, 49, 53, 63	
*Culiseta inornata*	6	42, 64–66	64, 65
*Psorophora ferox*	3	25, 31, 33, 34, 36, 41,^b^	
*Culex tarsalis*	6, 17, 19, 23, 24	25–27	64, 65, 69, 73–75
*Culex erraticus*		27–32	25, 33, 45, 51, 52, 59, 76, 77

^a^ Additional host use data included in Appendix 4.

^b^ L.E. Reeves, unpublished data.

## Appendix 3

Calculated enzootic and epizootic WNV vector potential.

All references from which data were extracted for the analysis are identified in Appendix 2, and data are included in the shared data. Meta‐analyses and vector potential calculations were conducted as described in the main manuscript.

**TABLE A3:** Enzootic and epizootic WNV vector potential of mosquito vector species in the Eastern Temperate Forests ecoregion of North America.

Species	Overall proportion			Vector potential	
	WNV vector competence	Avian host use	Mammal host use	Enzootic	Epizootic
*Psorophora ferox*	0.000	0.036	0.956	0.0000	00000
*Aedes japonicus*	0.646	0.000	1.000	0.0000	00000
*Aedes taeniorhynchus*	0.017	0.029	0.965	0.0005	0.0005
*Aedes canadensis*	0.029	0.019	0.817	0.0006	0.0005
*Aedes aegypti*	0.158	0.004	0.997	0.0006	0.0006
*Culiseta inornata*	0.214	0.007	0.994	0.0015	0.0015
*Coquillettidia perturbans*	0.022^a^	0.113	0.882	0.0025	0.0022
*Aedes cantator*	0.137	0.019	0.962	0.0026	0.0025
*Aedes vexans*	0.210	0.013	0.967	0.0027	0.0026
*Aedes sollicitans*	0.119	0.024	0.980	0.0029	0.0028
*Culex coronator*	0.076	0.089	0.911	0.0068	0.0062
*Aedes triseriatus*	0.251	0.047	0.950	0.0118	0.0112
*Aedes albopictus*	0.682	0.020	0.968	0.0136	0.0132
*Culex nigripalpus*	0.078	0.425	0.441	0.0332	0.0146
*Culex quinquefasciatus*	0.088	0.614	0.335	0.0540	0.0181
*Culex pipiens*	0.098	0.860	0.096	0.0843	0.0081
*Culex salinarius*	0.509	0.183	0.806	0.0931	0.0751
*Culex restuans*	0.108	0.907	0.076	0.0980	0.0074
*Aedes atropalpus*	0.917				

^a^ Vector competence as reported in Sardelis et al. (2001).

## Appendix 4

Supplementary analysis: Absolute VPI of select North American WNV vector species.

To compare WNV vector potential of *Culex tarsalis* with vector species of the Eastern Temperate Forests ecoregion, we quantified absolute VPI_10_ of these species (Table A4). *Culex tarsalis* vector competence and proportions of mosquito host use were calculated following the methods described in the main manuscript. Studies included in the supplementary analyses met all inclusion criteria outlined in the literature search section, except that for host use studies, the acceptable area of mosquito population origin was expanded to the contiguous United States. All references from which data were extracted for the analysis are identified in Appendix 2, and data are included in the shared data. Meta‐analyses were conducted as described in the main manuscript.

*Culex tarsalis* vector competence. Our literature search identified 5 published WNV vector competence studies on *Culex tarsalis* that met our inclusion criteria (Table A2). These studies resulted in 10 observations for inclusion in meta‐analyses (total sample size *N* = 790). A high amount of variance across the WNV vector competence data was not attributed to chance (*I* ^2^ = 94%), with differences within studies accounting for 92% of the explained variance. Such within‐study differences can include, for example, testing the vector competence of different mosquito populations in space or time, or different treatments in laboratory experiments.

Variance in host use data. A substantial amount of variance in the host use data set was not attributed to chance (*I* ^2^ = 99%). For both the avian and mammalian host use subset, this variance was largely explained by differences in the mosquito species investigated (*I* ^2^ = 53% and *I* ^2^ = 57%), while a lower amount of variance was explained by between‐study (*I* ^2^ = 35% and *I* ^2^ = 26%) and within‐study (*I* ^2^ = 12% and *I* ^2^ = 16%) differences, respectively.

**TABLE A4:** Absolute enzootic and epizootic WNV VPI of select mosquito vector species of North America.

Species	Host use			Overall proportion			Absolute VPI_10_	
	Ref	Obs	*N*	WNV vector competence	Avian host use	Mammal host use	Enzootic	Epizootic
*Psorophora ferox*	7	10	743	0.000	0.036	0.956	0	0
*Aedes japonicus*	2	2	60	0.646	0.000	1.000	0	0
*Aedes taeniorhynchus*	4	8	870	0.017	0.029	0.965	1	1
*Coquillettidia perturbans*	12	16	1435	0.022	0.113	0.882	1	1
*Aedes canadensis*	6	6	1685	0.029	0.019	0.817	1	1
*Culex coronator*	2	4	56	0.076	0.089	0.911	1	1
*Culex nigripalpus*	7	20	15992	0.078	0.425	0.441	1	1
*Culex quinquefasciatus*	21	36	5083	0.088	0.741	0.235	1	1
*Culex pipiens*	16	19	3786	0.098	0.879	0.088	1	1
*Culex restuans*	17	19	1993	0.108	0.907	0.076	1	1
*Aedes sollicitans*	7	7	1922	0.119	0.024	0.980	1	1
*Aedes cantator*	2	2	53	0.137	0.019	0.962	1	1
*Aedes aegypti*	3	5	283	0.158	0.004	0.997	1	1
*Aedes vexans*	17	24	5207	0.210	0.018	0.965	1	1
*Culiseta inornata*	4	5	1535	0.214	0.007	0.994	1	1
*Aedes triseriatus*	9	12	804	0.251	0.047	0.950	1	1
*Aedes albopictus*	7	9	1512	0.682	0.020	0.968	1	1
*Culex salinarius*	11	12	667	0.509	0.183	0.806	1	4
*Culex tarsalis*	8	19	14744	0.414	0.763	0.202	4	3
*Culex erraticus*	9	18	3356		0.357	0.411		

Abbreviations: N, total number of tested specimens; Obs, number of observations included in proportional meta‐analysis; Ref, number of identified studies.
